# A Combined Neutron and Synchrotron X‑ray Scattering Study of a MgAl-Layered Double Oxide

**DOI:** 10.1021/acs.inorgchem.5c03754

**Published:** 2025-11-10

**Authors:** Frederick Z.T. Yang, Theodosios Famprikis, Joerg Neuefeind, Mohsen Danaie, Claire T. Coulthard, Chunping Chen, Dermot O’Hare

**Affiliations:** a Chemistry Research Laboratory, Department of Chemistry, 6396 University of Oxford, 12 Mansfield Road, Oxford OX1 3TA, U.K.; b Inorganic Chemistry Laboratory, Department of Chemistry, 6396 University of Oxford, South Park Road, Oxford OX1 3QR, U.K.; c Neutron Scattering Division, 6146 Oak Ridge National Laboratory, Oak Ridge, Tennessee 37731, United States; d Electron Physical Science Imaging Centre, 120796 Diamond Light Source Ltd., Didcot OX11 0DE, U.K.

## Abstract

Owing to their vast chemical and structural flexibility, layered double hydroxides (LDHs) are among some of the most promising materials for many catalytic applications. Thermal decomposition below 700 °C leads to the formation of a complex semiamorphous mixed metal oxide (LDO). In this study, the product of calcination of aqueous miscible organic solvent-treated AMO-[Mg_0.70_Al_0.30_(OH)_2_]­(CO_3_)_0.15_·*y*H_2_O·*z*EtOH at 600 °C (AMO-Mg_2.33_Al LDO) has been investigated using a synergistic combination of high-resolution synchrotron X-ray and neutron scattering techniques, as well as high-angle annular dark-field scanning transmission electron microscopy (HAADF-STEM), solid-state NMR (ssNMR), and thermogravimetric analysis coupled with mass spectrometry (TGA-MS). The local and extended structure of AMO-Mg_2.33_Al LDO has been modeled by reciprocal and real space X-ray and neutron scattering analyses and is consistent with a modified rock salt structure consisting of octahedrally coordinated layers containing a small number of vacancies and the tetrahedrally coordinated Al^3+^ sites in contrast to previous reports.

## Graphical abstract

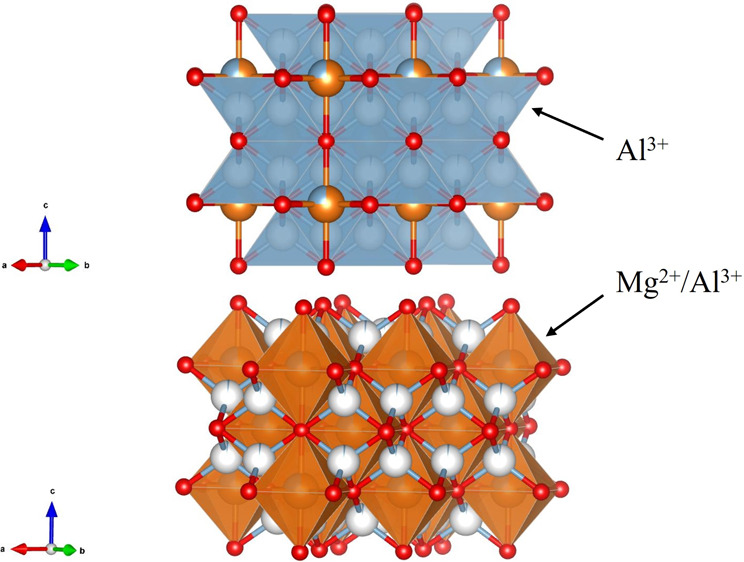

## Introduction

Layered double hydroxides (LDHs) are a class of lamellar compounds made up of positively charged brucite-like layers with an interlayer region containing negatively charged anions and water molecules. The most common formulation is expressed as [M^2+^_1–*z*_M′^3+^_*z*_(OH)_2_]­(A^*n*–^)_*z/n*_·*y*H_2_O; M_*x*_M′-A LDH; *x* = (1–*z)*/*z*))], where M^2+^ represents divalent metal cations, M^3+^ represents trivalent metal cations, and A^*n*–^ is the intercalated charge-balancing anion. Owing to their tunable composition and structural flexibility, LDHs have attracted significant interest in the fields of catalysis, electrochemistry, biomedicine, and environmental technologies.^1–5^ An important advance in their chemistry has been the preparation of highly dispersed and high surface area LDH via the so-called aqueous miscible organic solvent treatment (AMOST) technique.^6^ AMO-LDHs have a composition [M^2+^_1–*z*_M′^3+^_*z*_(OH)_2_]­(A^*n*–^)_*z*/*n*_·*y*H_2_O·*w*(solvent); AMO-M_*x*_M′-A LDH; *x* = (1–*z)*/*z*)]; it can be dispersed in nonpolar hydrocarbons and possess BET surface areas in excess of 400 m^2^ g^–1^; moreover, they are known to outperform conventional LDHs in many catalytic applications.^4,7^

Thermal decomposition of LDHs at elevated temperatures (400–700 °C) leads to the formation of mixed metal oxides or layered double oxides (LDOs) with relatively high specific surface areas and porosity.^8–14^ These properties are important for their ability to function as potential catalysts or catalyst supports. The crystal structure of [Mg_1–*z*_Al_*z*_(OH)_2_]­(CO_3_)_*z/2*_·*y*H_2_O; Mg_*x*_Al-CO_3_-LDHs at room temperature is well established as a layered *R*3*m* rhombohedral. Above 900 °C, the structure converts to the two thermodynamically stable phases, rock salt and spinel.^15–18^ However, there are many different reports concerning the crystal structure of LDOs prepared by the thermal decomposition of Mg_*x*_Al-LDHs at intermediate temperatures, 400–700 °C.^8–11,13,14,19,20^ Intriguingly, many Mg_*x*_Al LDOs are capable of recovering the parent rhombohedral structure upon contact with water and, optionally, with a charge-compensating anion. This property has been commonly referred to as the “memory effect”. The “memory effect” is widely used in different applications for the intercalation of anions with various natures and sizes into the interlayer spaces.^21^ However, there is no single answer to the question: why does the “memory effect” not work for all compositions. Previous studies suggest that the degree of recovery of a layered structure for Mg_*x*_Al-LDHs depends on the Mg^2+^ content.^10^

Different structural models for Mg_*x*_Al LDOs synthesized between 400 and 700 °C have been proposed in the literature. It has most commonly been described using the cubic *Fm*3̅*m* MgO salt-type structure with some incorporated Al^3+^.^13,22^ Two different cation defect rock salt structures have been proposed using Rietveld refinements of the X-ray diffraction data, Mg_0.57_Al_0.28_□_0.14_O and Mg_2(1–*x*)/(2+*x*)_Al_2*x*/(2+*x*)_□_(*x*/(2+*x*))_O (□ represents vacancies), depending on the Mg:Al ratio.^13,20^ Besides the single rock salt phase composition, Mg_*x*_Al-CO_3_ LDHs have also been proposed to decompose into rock salt and amorphous Al_2_O_3_.^23^

The main divergence of opinion concerns the extremely broad feature adjacent to the 220 Bragg reflection indexed to the *Fm*3̅*m* cubic space group. In PXRD data, this feature has often been indexed as the 111 Bragg reflection. However, upon Rietveld refinement, the calculated 111 Bragg reflection profile cannot fully account for this broad feature, indicating additional complexity in the crystal structure of the LDO.^20^ Diffuse scattering or the appearance of a spinel-like phase has been suggested in the literature.^9,10,20,22^

A spinel-like model with the *Fd*3̅*m* space group has also been considered due to the structural similarities between MgO and MgAl_2_O_4_ spinel. In this model, the rock salt oxide has been described using a supercell with a lattice parameter of a spinel with 32 oxygen atoms and 32 cations that fill all of the octahedrally coordinated positions. However, the octahedral sites are nonequivalent in relation to the rock salt *Fm*3̅*m* space group, and they are **16c** and **16d**. In a standard MgAl_2_O_4_, Al atoms are located in the **16c** site, and the **16d** site is empty, while the Mg atoms are located in the **8a** tetrahedral sites. This model produced a much better fit to the additional broad feature mentioned above than to the pure rock salt model.^22^

Previously, a “rock salt–spinel” intermediate-type structure has also been proposed in the literature with the cubic space group *Fd*3̅*m*, where each octahedral salt-type layer is alternated by a mixed spinel-like octahedra–tetrahedra layer. This model assumes that the octahedra rock salt-type layers are inherited from the parent LDH and the mixed spinel-like layers are formed between layers during dehydroxylation and carbonate decomposition. Rietveld refinement using this intermediate model gave a better fit than the pure rock salt model, producing an increase in calculated intensity of the 220 Bragg peak, and the broad region adjacent to the 111 Bragg reflection can also be accounted for using this model. The authors of this model attributed the broad feature to the presence of mixed spinel-like layers.^10,11^ A similar model has also been proposed for an Mg_*x*_Al LDO prepared from nitrate-intercalated Mg_*x*_Al-NO_3_ LDH where the structure consists of two kinds of layers.^19^ The first layer consists of rock salt-like octahedra filled with Mg^2+^, and the second layer is spinel-like with 25% octahedra and tetrahedra filled with Al^3+^. The authors attributed the broad peak to stacking disorder along the 111 direction.^19^

Given the wide-ranging application of these materials and the different structural models proposed in the literature, a more detailed and advanced characterization of the Mg_*x*_Al LDO structure is required. In this work, we have applied a combination of both synchrotron X-ray and neutron scattering techniques to probe the details of the LDO formed by calcination of AMO-[Mg_0.70_Al_0.30_(OH)_2_]­(CO_3_)_0.17_]·*y*H_2_O·*z*EtOH at 600 °C. Additional complementary characterization techniques, such as solid-state nuclear magnetic resonance spectroscopy (ssNMR), scanning transmission electron microscopy (STEM), thermogravimetric-mass spectrometry analysis (TGA-MS), and inductive coupled plasma (ICP) were used to support our structure analysis.

## Experimental Details

### Synthesis of AMO-[Mg_0.70_Al_0.30_(OH)_2_]­(CO_3_)_0.17_·*y*H_2_O·*z*EtOH; AMO-Mg_2.33_Al-LDH

100 mL of a metal precursor solution containing Mg­(NO_3_)_2_·6H_2_O (0.75 M) and Al­(NO_3_)_2_·9H_2_O (0.25 M) was added dropwise into 100 mL of 0.5 M [NH_4_]_2_(CO_3_). The pH value was kept at 10 by the dropwise addition of a 32% NH_3_ solution. After aging for 0.5 h with stirring at 750 rpm, the mixture was filtered and washed with DI water until the pH was close to 7. The resulting wet cake [Mg_0.70_Al_0.30_(OH)_2_]­(CO_3_)_0.15_·*x*H_2_O was further rinsed with ethanol (500 mL) followed by redispersion in 300 mL of ethanol with stirring at room temperature for 1 h. The resulting [Mg_0.70_Al_0.30_(OH)_2_]­(CO_3_)_0.15_·*y*H_2_O·*z*EtOH (AMO-Mg_2.33_Al-LDH) was isolated by filtration and dried in a vacuum oven at room temperature overnight.

### Synthesis of AMO-Mg_2.33_Al LDO

AMO-[Mg_0.70_Al_0.30_(OH)_2_]­(CO_3_)_0.17_·*y*H_2_O·*z*EtOH [AMO-Mg_2.33_Al-LDH] was calcined at 600 °C for 5 h at 10 °C/min to form AMO-Mg_2.33_Al LDO. The Mg_2.33_Al LDO used in the neutron diffraction experiment was synthesized from deuterated AMO-Mg_2.33_Al-LDH. This was prepared by dispersing AMO-Mg_2.33_Al-LDH in 200 mL of D_2_O and left at room temperature for 48 h, followed by drying in the vacuum oven at room temperature overnight. The deuterated AMO-Mg_2.33_Al-LDH was then calcined at 600 °C for 5 h to give the AMO-Mg_2.33_Al LDO.

### Characterization Techniques

High-resolution synchrotron powder X-ray diffraction (SXRPD) data were collected on the powder diffraction beamline, I11, at the Diamond Light Source (DLS). Silicon was used to determine the wavelength at 0.825960(4) Å. Samples were loaded into 0.5 mm glass capillaries. Room-temperature data were collected for the Mg_3_Al-LDH and LDO samples.

X-ray total scattering measurements were collected at the I15-1 beamline at the Diamond Light Source. The Mg_2.33_Al LDO sample was loaded into 1 mm glass capillaries, and the measurement was performed at room temperature with a collection time of 10 min. X-ray total scattering data were then transformed to the pair distribution function (PDF), *D*(*r*), via a sine Fourier transform. This transformation was performed using GudrunX.

Neutron diffraction data was also collected for the Mg_2.33_Al LDO sample at the TOF total scattering diffractometer NOMAD at the Spallation Neutron Source (SNS), Oak Ridge National Laboratory (ORNL). The LDO sample was ground and loaded into a 3 mm-diameter quartz capillary. Data was collected at 300 K for 1 h. Neutron total scattering data were then transformed to the pair distribution function (PDF), *G*(*r*). This transformation was performed with the in-house software at ORNL.

Structural refinement was performed using the Rietveld and PDF method implemented in software package TOPAS Academic (v6).^24^ PDF refinements were conducted using a fixed d*Q* and refined lattice parameters, scale, thermal parameters, and spherical dampening.

To quantitatively determine the crystallinity of the Mg_2.33_Al LDO phases, additional patterns were collected on samples that had been spiked with known quantities of crystalline silicon (of the order of 20 wt %). Multiphase Rietveld refinement including all phases was performed on these patterns, including LDO and silicon phases. Based on the difference between the refined silicon wt % and the known spiked wt %, the amorphous content of the sample was determined according to the following equations.

$$W_{\mathrm{LDO}}=\frac{W_{\mathrm{Si},\mathrm{Known}}}{W\prime_{\mathrm{Si}}}\times W\prime_{\mathrm{LDO}}\times 100\%$$ 1

$$W_{\mathrm{amorphous}}=100\%-W_{\mathrm{LDO}}-W_{\mathrm{Si},\mathrm{Known}}$$ 2

where, *W*_Si,known_ is the spiked weight percent of silicon in the sample and *W*′_Si_ is the apparent weight percent of silicon from the Rietveld refinement. *W*_LDO_ is the true and apparent weight percentages of crystalline LDO. *W*_amorphous_ is the weight percent of the sample that is not crystalline, which is assumed to come solely from the amorphous content of LDO. Based on this analysis, the percent crystallinity of the LDO can be determined.

Transmission electron microscopy (TEM) images were obtained with a JEOL 2100 microscope using an acceleration voltage of 200 kV. Scanning transmission electron microscopy images were acquired on a JEOL ARM300F microscope at the electron Physical Science Imaging Centre (ePSIC), using an acceleration voltage of 300 kV, a convergence semiangle of 26.2 mrad, and a beam current of 25 pA. ^27^Al solid-state nuclear magnetic resonance spectroscopy (ssNMR) was obtained on a Bruker Advance III HD Solid State NMR equipped with a 9.4 T magnet in 4.0 mm O.D zirconia rotors.

Thermogravimetric-mass spectrometry analysis (TGA-MS) was carried out using a PerkinElmer TGA 8000. Weight change under air and the evolution rates of H_2_O, CO_2_, and ethyl alcohol (EtOH) from 30 to 900 °C were measured for the AMO-LDH sample with a 10 °C min^–1^ ramp rate. Inductive coupled plasma (ICP-OES) was used to determine the Mg and Al composition in AMO-LDH and AMO-LDO samples.

## Results and Discussion

### Synthesis

[Mg_0.70_Al_0.30_(OH)_2_]­(CO_3_)_0.17_·*x*H_2_O was prepared by coprecipitation at pH 10 using the respective metal nitrate salt ratios in the presence of an excess of [NH_4_]_2_(CO_3_). The neutralized LDH wet cake was resuspended in dry ethanol to give a highly dispersed, high surface area AMO-[Mg_0.70_Al_0.30_(OH)_2_]­(CO_3_)_0.15_·*y*H_2_O·*z*EtOH (AMO-Mg_2.33_Al-CO_3_ LDH) suspension, which was then filtered and dried under vacuum. ICP was used to confirm the Mg:Al ratio at 2.33. The H_2_O and EtOH (*y* + *z*) content was determined from the TGA data.

### Thermal Decomposition of AMO-Mg_2.33_Al-CO_3_ LDH

The thermal decomposition of carbonate-containing LDHs commonly occurs by three thermal events.^25,26^ In the case of AMO-LDHs, there is an additional common feature of the AMO solvent (e.g., EtOH) desorption. The thermogravimetric analysis (TGA) and differential curve (dTGA) for AMO-Mg_2.33_Al-CO_3_ LDH and the gas evolution profiles for H_2_O, CO_2_, and EtOH is shown in Figure 1a and Figure 1b, respectively.

**Figure 1:**
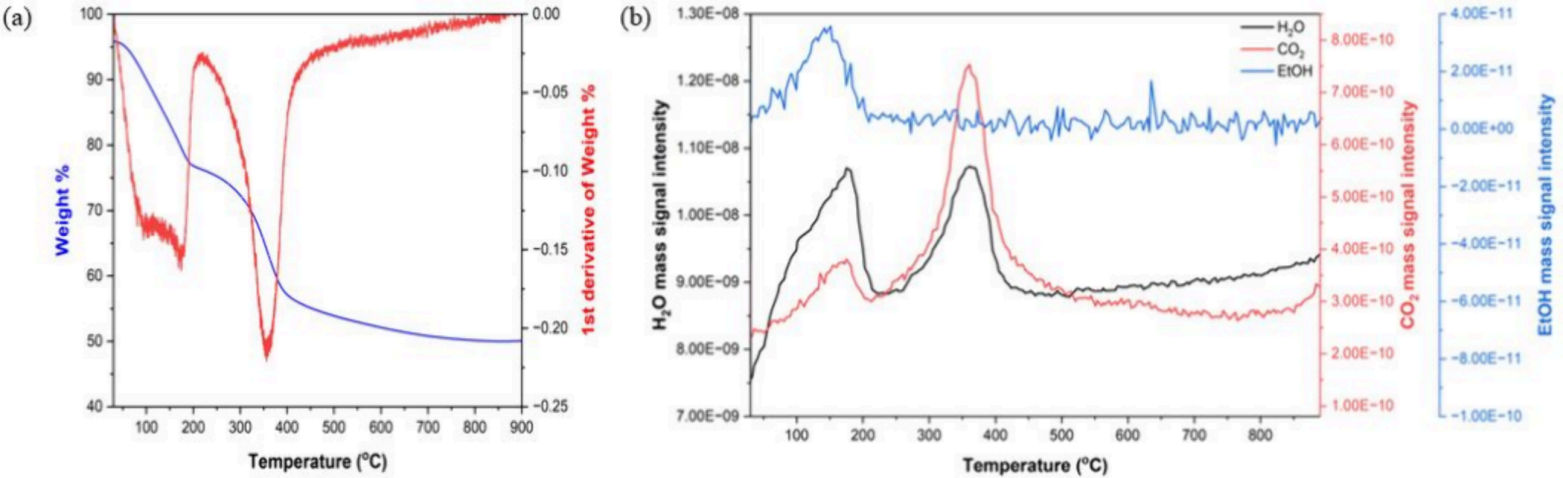
(a) TGA-DTG curves and (b) H_2_O, CO_2_, and EtOH evolution from AMO-Mg_2.33_Al-CO_3_ LDH upon heating from 30 to 900 °C.

The removal of both surface-bound water and surface-bound EtOH starts below 100 °C. Complete removal, including any interlayer water, occurs below 200 °C. From the weight loss below 200 °C, we estimate the sum of *y* and *z* to be *ca*. 0.73. Dehydroxylation and interlayer carbonate decomposition occur around 360 °C. It is interesting to note that the typical temperature reported in the literature for the final third step is around 400 °C for crystalline LDHs.^9,10,26,27^ The lower decomposition temperature seen here for AMOST-treated LDH is a common feature and has been attributed to the better dispersion (N_2_ BET = 414 m^2^/g) and a thinner platelet morphology of the AMO-LDH particles.^6^

### Bragg Synchrotron X-ray, Neutron Diffraction, and ^27^Al ssNMR Studies of AMO-Mg_2.33_Al-CO_3_ LDH

The average structure of the AMO-Mg_2.33_Al-CO_3_ LDH was found to adopt the rhombohedral *R*3̅*m* space group by using Rietveld refinement of the SXRPD data (Figure S1). This agrees with previous studies of AMO-Mg_*x*_Al-LDHs.^9,13,15^ In addition, the ^27^Al ssNMR data of AMO-Mg_2.33_Al-CO_3_ LDH showed a single sharp resonance at 8.19 ppm, indicating the presence of Al^3+^ ion in only octahedral sites (Figure S2a).^28^

The AMO-Mg_2.33_Al-CO_3_ LDH was then transformed to the layered double oxide (AMO-Mg_2.33_Al LDO) upon calcination at 600 °C in air (5 h, 10 °C/min). Rietveld refinement of the SXRPD of the AMO-Mg_2.33_Al LDO sample was initially fitted with a rock salt *Fm*3̅*m* cubic MgO model (*R*_wp_ 3.29%). The calculated intensity of the 220 Bragg peak is slightly lower than the experimental one, indicating the presence of additional components in the crystal structure (Figure 2a). A Fourier difference map was calculated on the refinement, which suggested the presence of an additional atom with a tetrahedra coordination at the 14 , 14 , 14 site between the octahedrally coordinated metal layers (Figure S3). Due to the similar X-ray scattering factors of Mg and Al, it is difficult to determine the identity of this tetrahedrally coordinated atom from Rietveld refinement alone. The ^27^Al ssNMR data for LDO calcined at 600 °C showed the presence of both octahedrally and tetrahedrally coordinated Al^3+^ with the appearance of an additional peak at 72.90 ppm (Figure S2b); this agrees with previous ssNMR studies of Mg_*x*_Al LDOs.^28^ The integration of two ssNMR peaks revealed that 55% of Al^3+^ is tetrahedrally coordinated and 45% remains in an octahedral site. This indicates the partial transition of Al^3+^ ions that were previously in the octahedral sites into the tetrahedral sites after calcination at 600 °C and the presence of possible octahedral-site vacancies.

**Figure 2:**
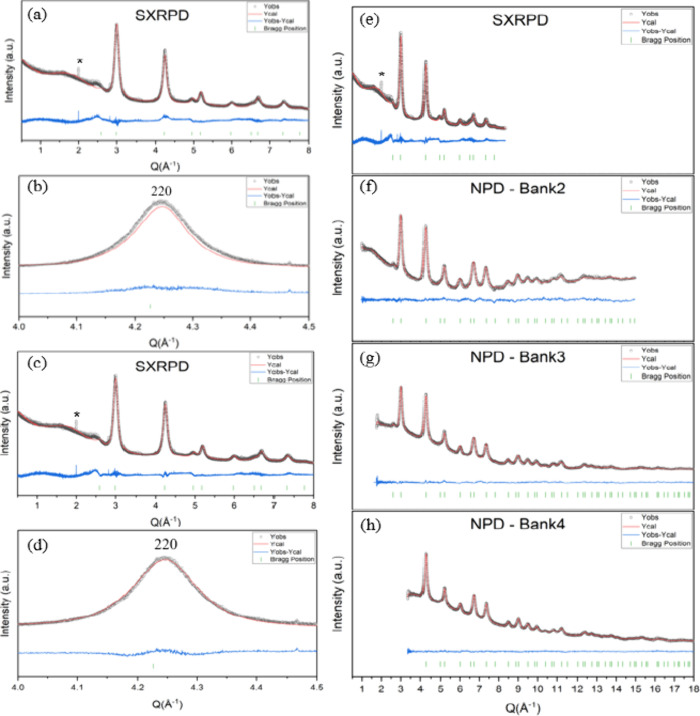
Rietveld refinement profile of SXRPD and NPD data of AMO-Mg_0.70_Al_0.30_ LDO using TOPAS (v6). (a) Pure rock salt model without the tetrahedrally coordinated Al and (b) enlargement of the 220 peak. (c) Rock salt model with the tetrahedrally coordinated Al and (d) enlargement of the 220 peak. (e–h) Refined SXRPD and NPD data with the inclusion of tetrahedrally coordinated Al as a function of *Q* (Å^–1^). (*) indicates a detector error occurred during data collection.

The calculated intensity of the 220 Bragg peak improved with the addition of Al in the 14 , 14 , 14 site in the refinement along with a decrease in *R*_wp_ to 2.94% (Figure 2c,d). This shows substantial modifications in the brucite layers of the LDH upon decomposition, with the migration of some Al^3+^ to tetrahedral sites in the interlayers. Refined parameters from SXRPD can be found in Table S1.

Neutron Bragg diffraction data collected using the NOMAD instrument was initially fitted with the rock salt *Fm*3̅*m* cubic MgO model (*R*_wp_ 0.85%), and a lower calculated intensity was similarly observed for the 220 reflection. The fit to the neutron data also improved with the addition of Al in the 14 , 14 , 14 site (*R*_wp_ 0.77%), consistent with preceding with SXRPD data analysis. Given the larger differences in neutron scattering lengths between atoms for neutron diffraction, the occupancies of each atom were refined (Table S1).

Refinement of the neutron diffraction data determined the Al^3^^+^ site occupancies to be 0.13(4) in the octahedral positions and 0.07(5) in the tetrahedral positions; this corresponds to 49% of Al^3^^+^ ions in octahedral coordination and 44% in tetrahedral coordination. These values are in good agreement with the distribution obtained from the integration of ^27^Al ssNMR data, falling within the experimental margin of error. A key finding is the presence of cation vacancies in the structure. The material’s stoichiometry (Mg_2_._3__3_Al, normalized to Mg_0_._7_Al_0_._3_) dictates a theoretical Al^3^^+^ occupancy of 0.3. The experimentally determined occupancy in the octahedral sites (0.13) is significantly lower, indicating a substantial vacancy concentration of 0.17 at these positions. It is interesting to note that the refined thermal parameter *B*_iso_ of the tetrahedrally coordinated Al^3+^ in both SXRPD and NPD are uncharacteristically high (1.75(1) Å^2^ and 3.95(1) Å^2^, respectively) compared to the octahedrally coordinated cations (0.25(3) Å^2^ and 0.39(1) Å^2^, respectively), indicating positional instability and weak interlayer bonding. This can potentially explain the “memory effect” seen in Mg_*x*_Al-A LDHs where the initial LDH structure is recovered from the corresponding LDO upon contact with water as the weakly tetrahedrally bonded Al^3+^ cations can migrate back to their original octahedral sites.^21^ The occupancies of Mg^2+^ and O^2–^ were found to be 0.68(4) and 0.98(2), respectively, within error of the chemical composition. Finally, a combined refinement of both SXRPD and NPD was conducted; this also yielded similar results to the individual refinements (Figure 2e–h and Table S1).

Attempts were made to fit the broad feature next to the 111 reflection (*Q* = 2.46 Å^–1^) seen in SXRPD (Figure 2) using models that have been previously suggested in the literature such as the spinel-like model^10,22^ (Figure S4), the “rock salt–spinel” intermediate model (Figure S5),^19^ and the addition of a second Al_2_O_3_ phase (Figure S6).^23^ These models were indeed able to account for the broad feature; however, it is difficult to know which model is more reliable based on the goodness of fit as one can fit multiple peaks under the single broad peak. In addition, these fits also produced unrealistic thermal parameters. The appearance of a broad peak indicates the noncrystalline nature of LDO and the presence of amorphous content. Quantitative phase analysis of LDO measured with an internal Si standard reveals that 3.8% of the sample is amorphous. The appearance of the broad feature could be due to the noncrystalline nature rather than the presence of amorphous content in the sample.

### Investigation of the Short-Range Structure of AMO-Mg_2.33_Al LDO Using Pair Distribution Function Analysis

Pair distribution function (PDF) methods have been applied previously to analyze the structure of LDHs and their thermal decomposition product up to 450 °C.^29–32^ However, to the best of our knowledge, the combination of synchrotron and neutron PDF techniques has not been previously applied to LDOs calcined at 600 °C. The Rietveld analysis of synchrotron and neutron powder diffraction patterns in the previous section provided detailed insights into the long-range structure of AMO-Mg_2.33_Al LDO calcined at 600 °C. Pair distribution function (PDF) data are sensitive to local-scale atom correlations, which are not visible in the long-range structure. Synchrotron X-ray and neutron total scattering data were collected to investigate the crystal structure on the local scale.

The first step in the analysis of PDF data was visual inspection. It can be seen that the intensity of the PDF peaks in both sets of data diminishes around 25 Å (Figure S7), indicating the nanoscopic nature of the material. The *r* ≤ 4.5 Å region indicates the short-range correlations for the nearest neighbors and the next nearest neighbor’s interatomic distances in a standard ∼4 Å cell (Figure 3a). The first peak represents the Mg/Al–O interatomic distance at 2.07 Å, and the next two peaks represent the Mg/Al–Mg/Al at 2.97 Å and Mg/Al–O at 3.62 Å, respectively, for a typical MgO rock salt-type structure. An additional shoulder peak can also be seen at 1.80 Å next to the first Mg/Al–O peak in the XPDF; this indicates possible displacements of the metal cations from the octahedral site and the presence of a new bonding environment.

**Figure 3:**
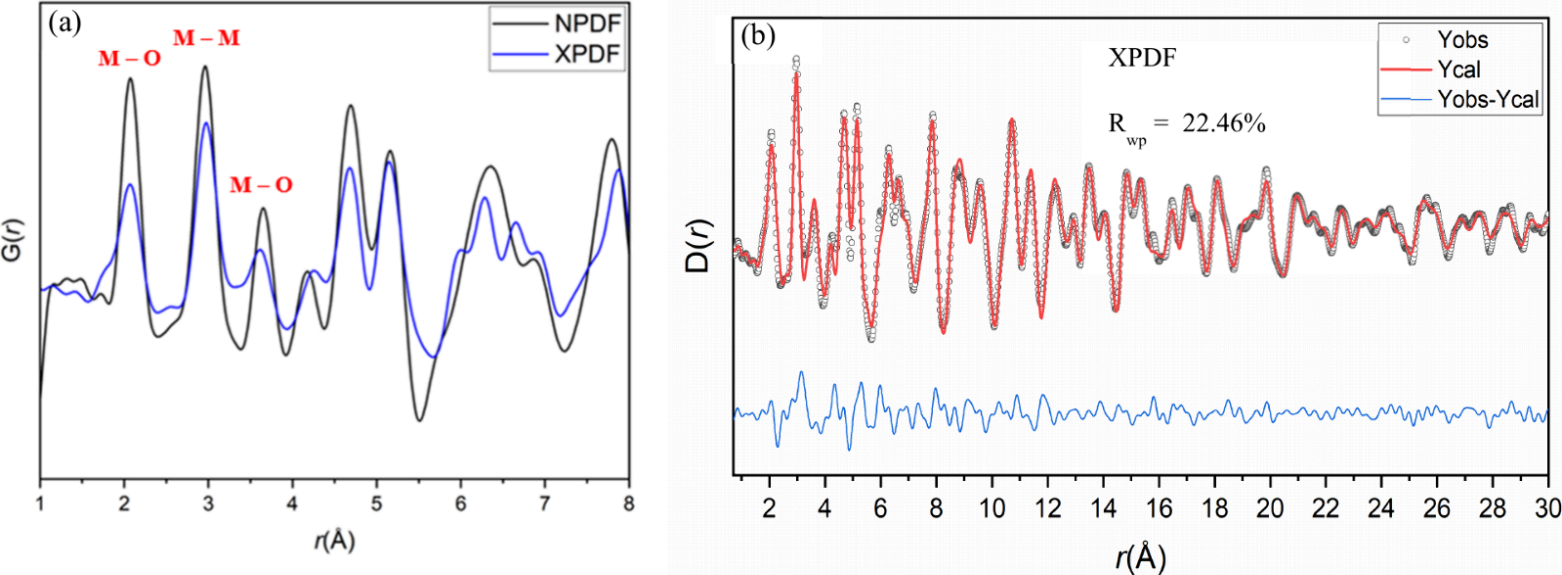
(a) NPDF and XPDF data of AMO-Mg_2.33_Al LDO between 1 and 8 Å. Red markers have been included to indicate peaks of the nearest neighbor atom–atom pairs in a rock salt structure. (b) Small-box PDF modeling of AMO-Mg_0.70_Al_0.30_ LDO XPDF in the range of 1–30 Å using the *Fm*3̅*m* rock salt model with tetrahedrally coordinated Al^3+^.

Small-box modeling (or real space Rietveld) was conducted on both the X-ray and neutron PDF from 1 to 30 Å using structural parameters from Rietveld refinement as a starting point without the tetrahedrally coordinated Al. The rock salt *Fm*3̅*m* model gave an adequate fit to both PDF patterns of LDO calcined at 600 °C. However, the pure *Fm*3̅*m* rock salt model cannot account for the shoulder peak at 1.80 Å seen in XPDF (Figure 4a). This shoulder peak could only be accounted with the inclusion of Al in the 14 , 14 , 14 position with the *R*_wp_ lowering from 27.77 to 22.46% in the XPDF (Figure 4a,b). This agrees with the displacement of the Al^3+^ cation from the octahedral sites to the tetrahedral sites discussed previously. We find an agreement with the Bragg data discussed earlier at a 30 Å length scale using the same structural model (Figure 3a and Figure S8). Detailed refined structural parameters are summarized in Table S1.

**Figure 4:**
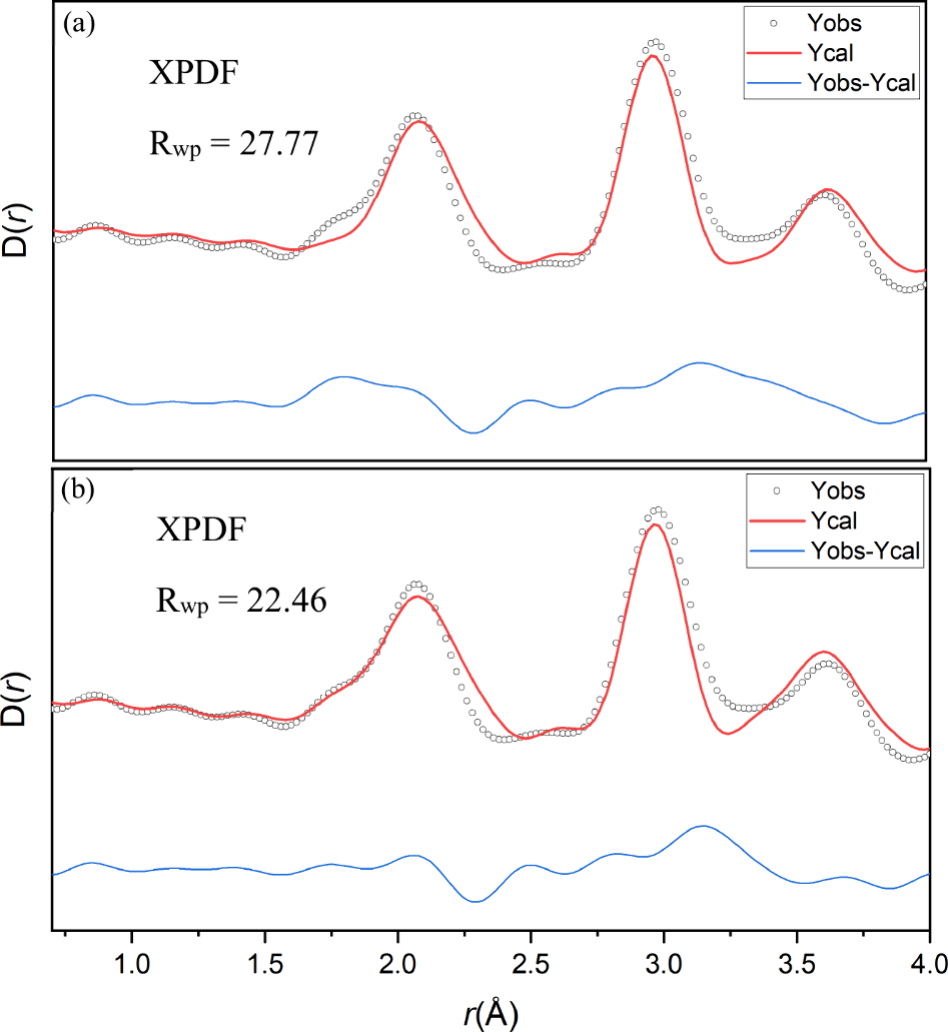
Small-box PDF modeling of AMO-Mg_2.33_Al LDO of X-ray PDF data between 1 and 4 Å without the tetrahedrally coordinated Al (a) and with the tetrahedrally coordinated Al (b).

The conjoint XPDF and NPD refinement showed that all of the individual and joint fits converge to the same solution with negligible deviation (Table S1). Attempts were also made to fit both XPDF and NPDF using models that have been previously suggested in the literature such as the spinel-like model,^22^ the “rock salt–spinel” intermediate model,^10^ and the addition of a second Al_2_O_3_ phase.^23^ A two-phase fit with a pure rock salt + Al_2_O_3_ and the spinel-like both yielded a better overall fit with lower *R*_wp_ values of 19.16 and 18.01%, respectively; however, it also produced unrealistic TOPAS-specific thermal parameters for Al_2_O_3_ (Figures S9 and S10). The “rock salt–spinel” intermediate model gave the worst fit, with a *R*_wp_ value of 32.47% (Figure S11). The diffuse feature seen in Bragg data discussed in the previous section likely originates from local correlated disorder that would not be captured by the small-box models (e.g., Rietveld) used here, so it is not entirely surprising that the diffuse feature is not reproduced.

### Study of the Morphology of AMO-Mg_2.33_Al LDO

The morphology of AMO-Mg_2.33_Al LDO was examined using transmission electron microscopy (TEM). Figure 5a shows that the sample is composed of flower-type particles constituted of corrugated nanosheets. This corresponds well with the low crystallinity observed in Bragg SXRPD and NPD as there is no distinct stacking order and the diameter of the particles is 120–150 nm. This is also consistent with the morphology of AMOST-treated samples.^6,33^ In addition, it can be seen that after calcination at 600 °C, the particles became thinner and smaller nanosheets compared to the previously reported AMOST-treated Mg_*x*_Al-LDH.^6,34^ High-angle annular dark-field scanning transmission electron microscopy (HAADF-STEM) was employed to directly visualize the atomic distribution within each nanosheet.

**Figure 5:**
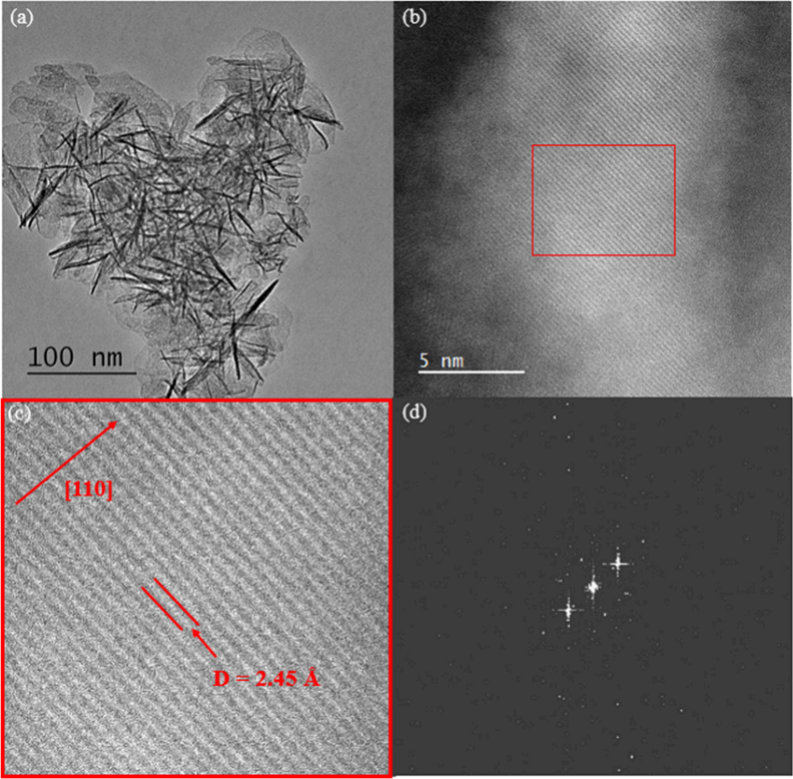
(a) TEM image and (b) HAADF-STEM image of AMO-Mg_2.33_Al LDO. (c) Enhanced section of a dark-field image showing the layered characteristic of LDO with an interlayer distance of 2.45 Å. (d) Fast Fourier transform of the enhanced section of the dark-field image.

The dark-field image clearly reveals a layered arrangement within each nanosheet (Figure 5b,c). This indicates that the structure has preserved the layered characteristic of LDH in the LDO following thermal decomposition. Fast Fourier transform was applied to the enhanced section of the dark-field image, and the *d* spacing was found to be 2.45 Å (Figure 5d) along the [110] direction. This is similar to the distance obtained from the Bragg and PDF analyses discussed above.

### Proposed Structure for AMO-Mg_2.33_Al LDO

We propose a model for AMO-Mg_2.33_Al LDO by combining high-resolution X-ray and neutron Bragg and PDF diffraction analyses with microscopy and ssNMR. The model is based on a modified rock salt MgO structure (Figure 6). The structure contains octahedrally coordinated rock salt layers occupied by Mg^2+^ and Al^3+^ (Figure 6b) together with tetrahedrally coordinated Al^3+^ located between the octahedral layers (Figure 6c). Vacancies are proposed in both octahedral and tetrahedral sites due to the migration of Al^3+^ during thermal decomposition and the partial occupancies of Al^3+^ between the octahedral layers. The chemical composition obtained from refinement of diffraction data is (Mg_0.68_Al_0.1_□_0.17_)_oct_(Al_0.07_)_tet_O_0.96_ (□ represents vacancies) and is within error of the metal content obtained from ICP Mg_0.71_Al_0.29_O. The modeling of PDF data confirmed the absence of any spinel-like characteristic for AMO-Mg_2.33_Al LDO, which had been previously suggested in the literature.^10,13,19,22^

**Figure 6:**
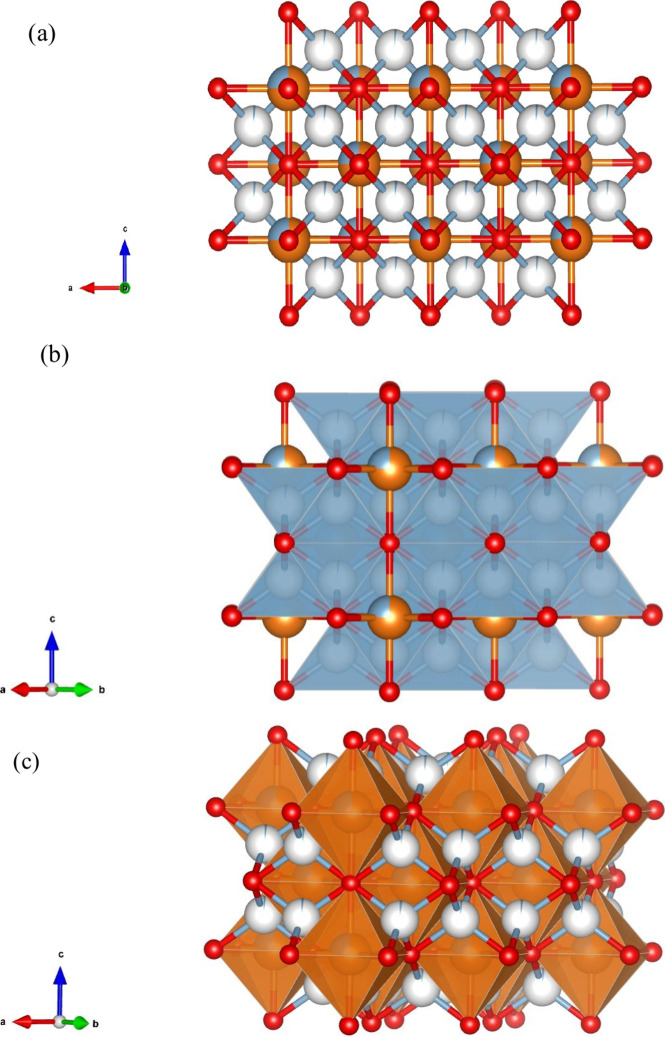
(a) Representation of the crystal structure of AMO-Mg_2.33_Al LDO obtained from refinement of X-ray and neutron Bragg and PDF diffraction data. Mg atoms in orange, Al in light blue, and O atoms in red. (b) Figure highlighting layers of the Mg/Al octahedra and (c) figure highlighting the tetrahedrally coordinated Al layers. (A CIF file of this model has been uploaded to CCDC, deposition number: 2491547).

## Conclusions

The AMO-Mg_2.33_Al LDO was synthesized from AMO-Mg_2.33_Al-LDH using the coprecipitation method, and the crystal structure was determined using X-ray and neutron scattering data collected at room temperature. AMO-Mg_2.33_Al LDO was found to be a modified rock salt *Fm*3̅*m* structure with tetrahedrally coordinated Al^3+^ between the octahedrally coordinated Mg^2+^ and Al^3+^ cations.

We hope that this accurate structure determination of this complex class of material will enable a better understanding of the structure–property relationship in mixed metal oxides and facilitate targeted applications through chemical and physical modifications.

## Supplementary Material



## Notes

Additional characterization data and methods (PDF)
