# Supplementary material for: A Combined Neutron and Synchrotron X‑ray Scattering Study of a MgAl-Layered Double Oxide
Source: Inorg Chem. 2025 Nov 10;64(46):22720–8. doi: 10.1021/acs.inorgchem.5c03754 (PMC12648650; doi:10.1021/acs.inorgchem.5c03754)
Supplement: Supplementary file 1 [file ic5c03754_si_001.pdf]

Supplementary Data

A combined neutron and synchrotron X-ray scattering study of a MgAl layered double oxide

Frederick Z.T. Yang[a], Theodosios Famprikis[b], Joerg Neuefeind[c], Mohsen Danaie[d], Claire T. Coulthard[a], Chunping Chen[a] and Dermot O'Hare[a],

[a] *Chemistry Research Laboratory, Department of Chemistry, University of Oxford, 12 Mansfield Road, Oxford, OX1 3TA, UK*

[b] *Inorganic Chemistry Laboratory, Department of Chemistry, University of Oxford, South Park Road, Oxford, OX1 3QR, UK*

[c] *Neutron Scattering Division, Oak Ridge National Laboratory, Oak Ridge, Tennessee, 37731, USA*

[d] *electron Physical Science Imaging Centre, Diamond Light Source Ltd., Didcot, OX11 0DE, UK*

**Corresponding author: dermot.ohare@chem.ox.ac.uk*

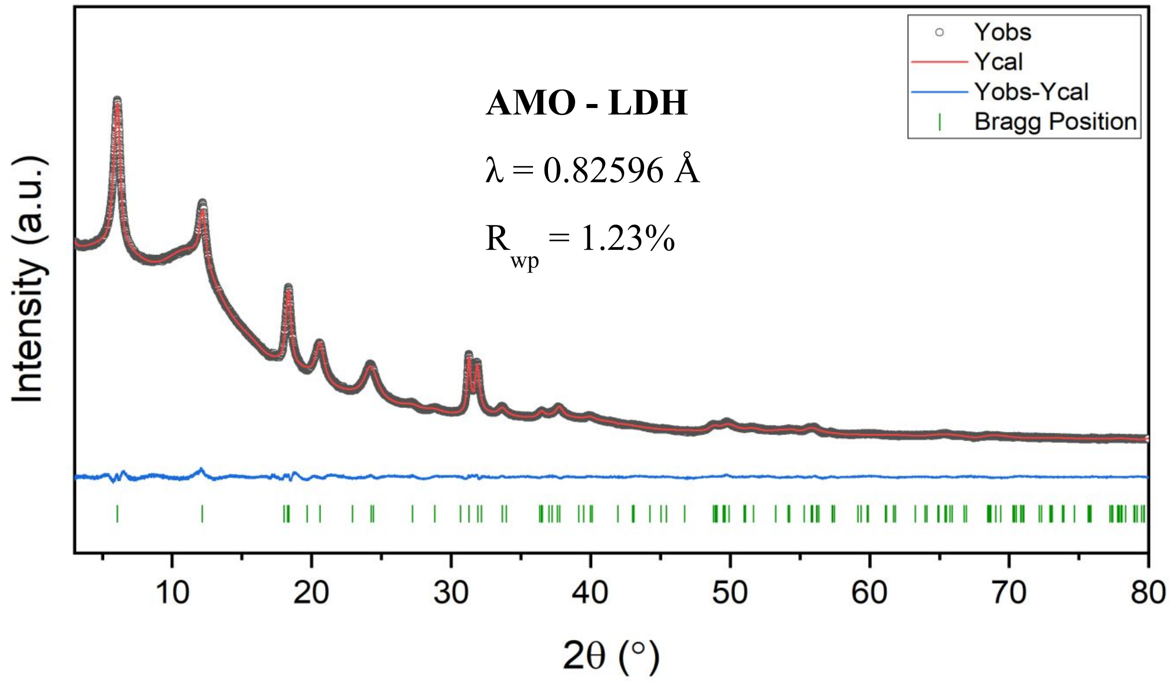

Figure S1. Rietveld refinement profile of S-XRPD data of AMO-$Mg_{2.33}Al$ LDO LDH using TOPAS (v6).

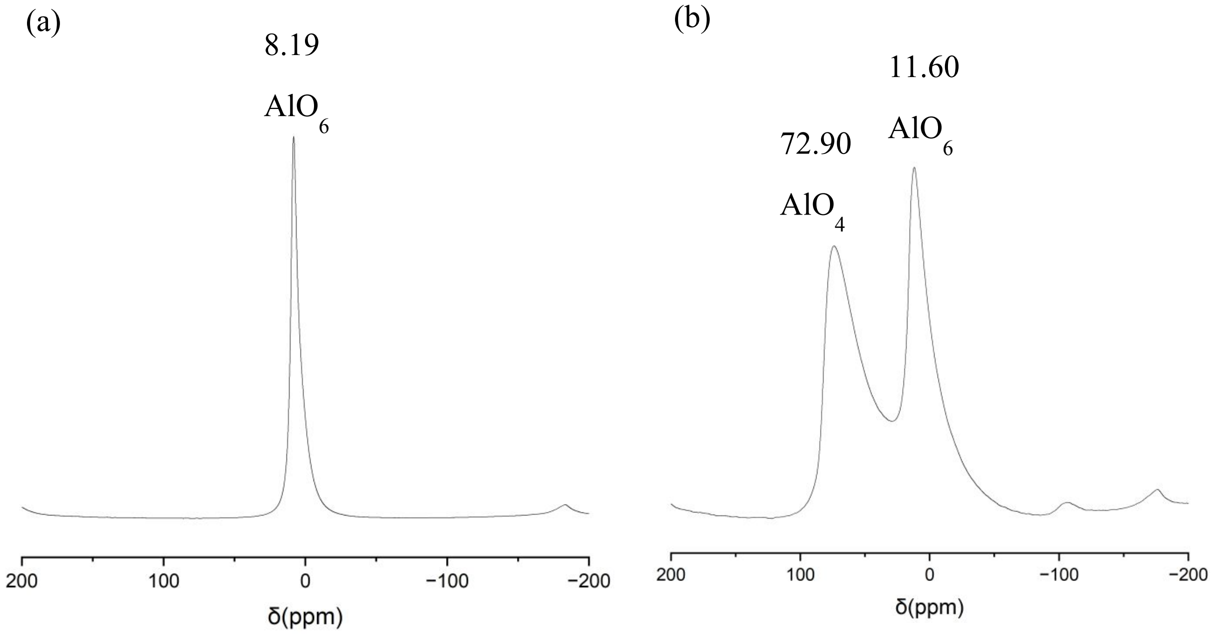

Figure S2. ^{27}Al ssNMR spectra of (a) AMO-$Mg_{2.33}Al$ LDH and (b AMO-$Mg_{2.33}Al$ LDO.

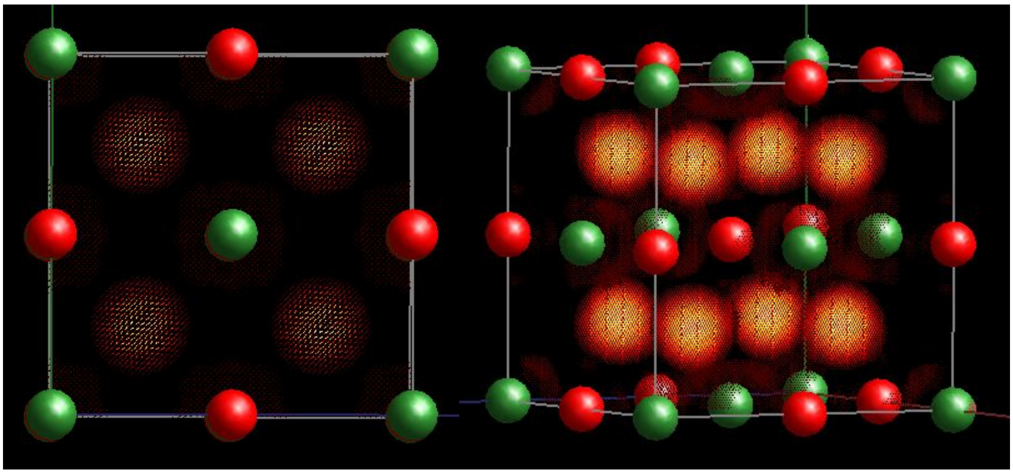

Figure S3. Fourier difference map of the AMO-$Mg_{2.33}Al$ LDO of the SXRPD data showing the presence of missing electron density at the ¼, ¼, ¼, position. The strategy implemented to find the tetrahedrally coordinated Al^{3+} positions from Fourier maps was to first fit a model containing only Mg, Al, and O atoms on positions defined in the literature *Fm*3*m* model. Peak shape profile terms, cell parameters, scale factors and background terms were allowed to refine. Clear positive scattering differences can be found at the ¼, ¼, ¼, position between the octahedrally coordinated rock salt layers.

Table S1. Refined structural parameters of AMO-$Mg_{2.33}Al$ LDO from SXRPD, NPD, XPDF, and NPDF

	SXRPD	NPD	Combined SXRPD + NPD	XPDF	NPDF	Combined XPDF + NPDF
**Space Group**	$Fm\bar{3}m$	$Fm\bar{3}m$	$Fm\bar{3}m$	$Fm\bar{3}m$	$Fm\bar{3}m$	$Fm\bar{3}m$
**R_{wp}%**	2.94	0.76	1.68	20.03	21.06	22.19
**a (Å)**	4.210866(5)	4.180974 (1)	4.211789(4)	4.202896(3)	4.194998(2)	4.19365(1)
**Volume (Å^3)**	74.665(2)	73.086(6)	74.714(2)	74.241(2)	73.824(1)	73.896(6)
**Mg_x/$Al1_x$**	0	0	0	0	0	0
**Mg_y/$Al1_y$**	0	0	0	0	0	0
**Mg_z/$Al1_z$**	0	0	0	0	0	0
**Mg occ**	0.7	0.68132(4)	0.69008(6)	0.68132	0.65207(2)	0.67802(2)
**Al1 occ**	0.135	0.13358(4)	0.13863(4)	0.11691	0.12118(3)	0.13511(3)
**B_{iso}(Å^2)**	0.25369(3)	0.39219((1)	0.51709(1)	-	-	-
**$Al2_x$**	1/4	1/4	1/4	1/4	1/4	1/4
**$Al2_y$**	1/4	1/4	1/4	1/4	1/4	1/4
**$Al2_z$**	1/4	1/4	1/4	1/4	1/4	1/4
**Al2 occ**	0.0825	0.07181(5)	0.07998(5)	0.07181	0.08248(1)	0.06891(4)
**B_{iso}(Å^2)**	1.75191(1)	3.95095(1)	2.30697(5)	-	-	-
**O_x**	1/2	1/2	1/2	1/2	1/2	1/2
**O_y**	1/2	1/2	1/2	1/2	1/2	1/2
**O_z**	1/2	1/2	1/2	1/2	1/2	1/2
**O occ**	1	0.98124(2)	0.99518(1)	0.98124	0.96253(2)	0.96253
**B_{iso}(Å^2)**	1.71555(2)	0.56089(2)	0.51440(1)	-	-	-

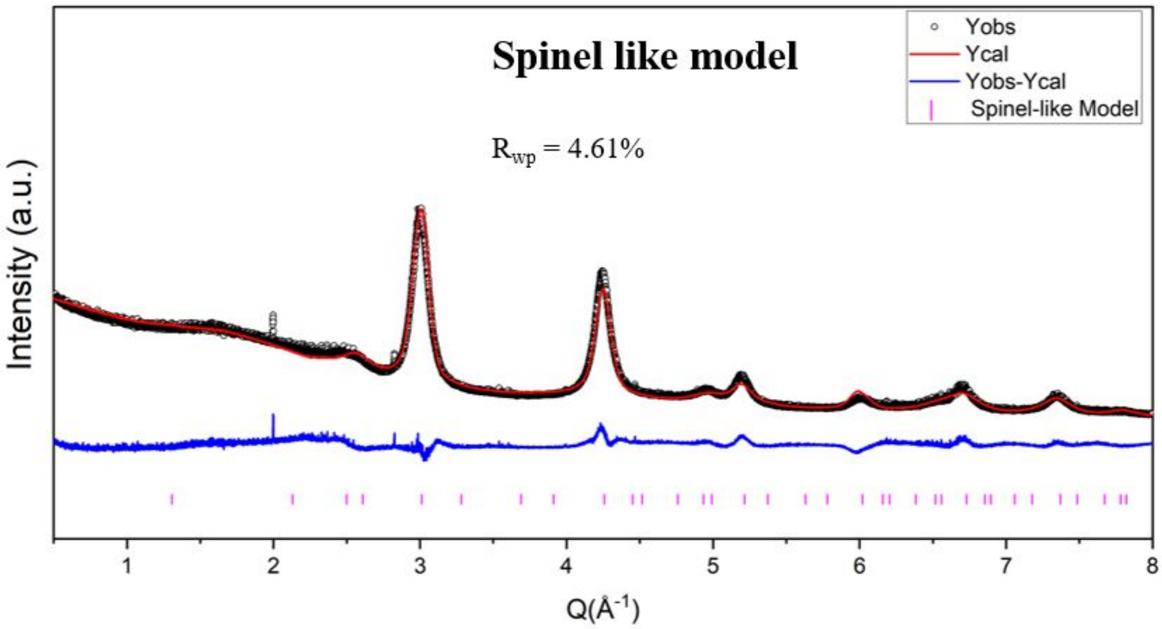

Figure S4. Rietveld refinement of AMO-$Mg_{2.33}Al$ LDO SXRPD data using a modified spinel model proposed by Cherepanova *et al.*[1]

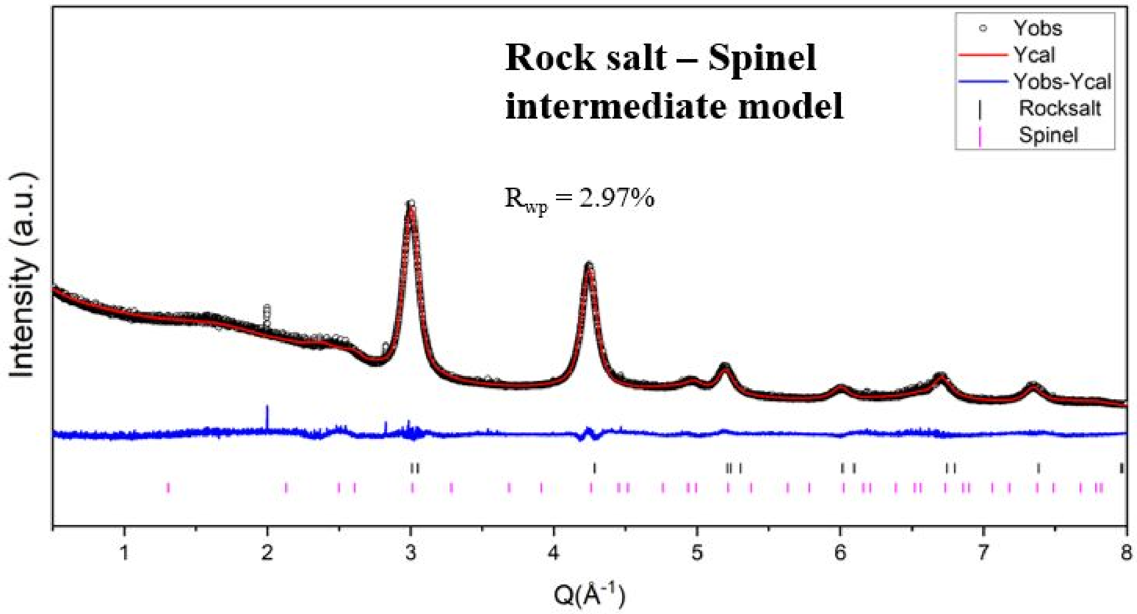

Figure S5. Rietveld refinement of AMO-$Mg_{2.33}Al$ LDO SXRPD data using a Rock salt – spinel intermediate model proposed by Johnsen *et al.*[2]

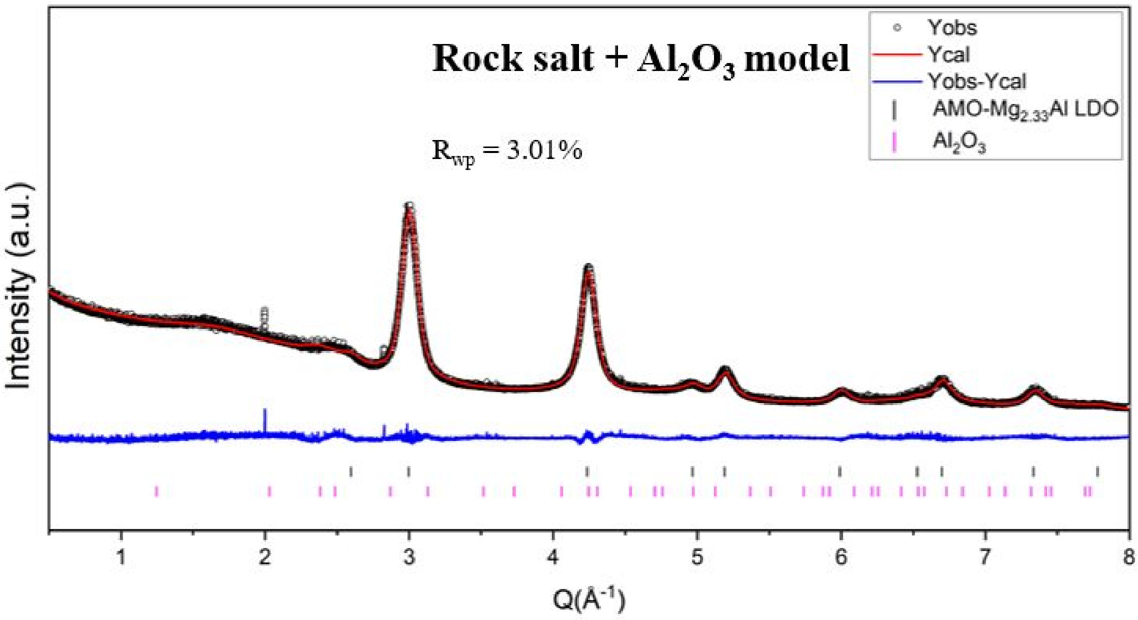

Figure S6. Rietveld refinement of AMO-$Mg_{2.33}$Al LDO SXRPD data using a two phase Rock salt and Al_2O_3 model proposed by Aramendia *et al.*[3]

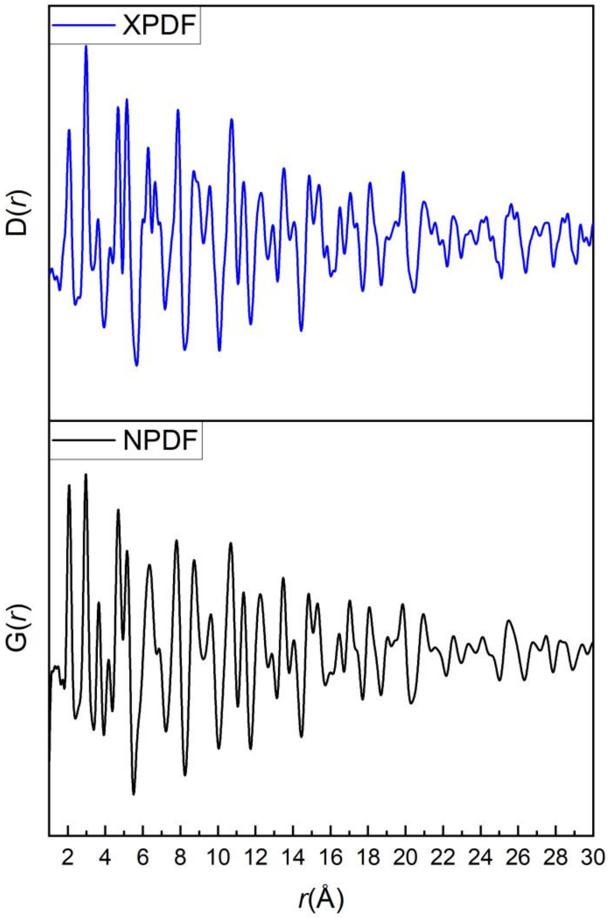

Figure S7. Raw XPDF and NPDF patterns of AMO-$Mg_{2.33}Al$ LDO SXRPD between 1 – 30 Å.

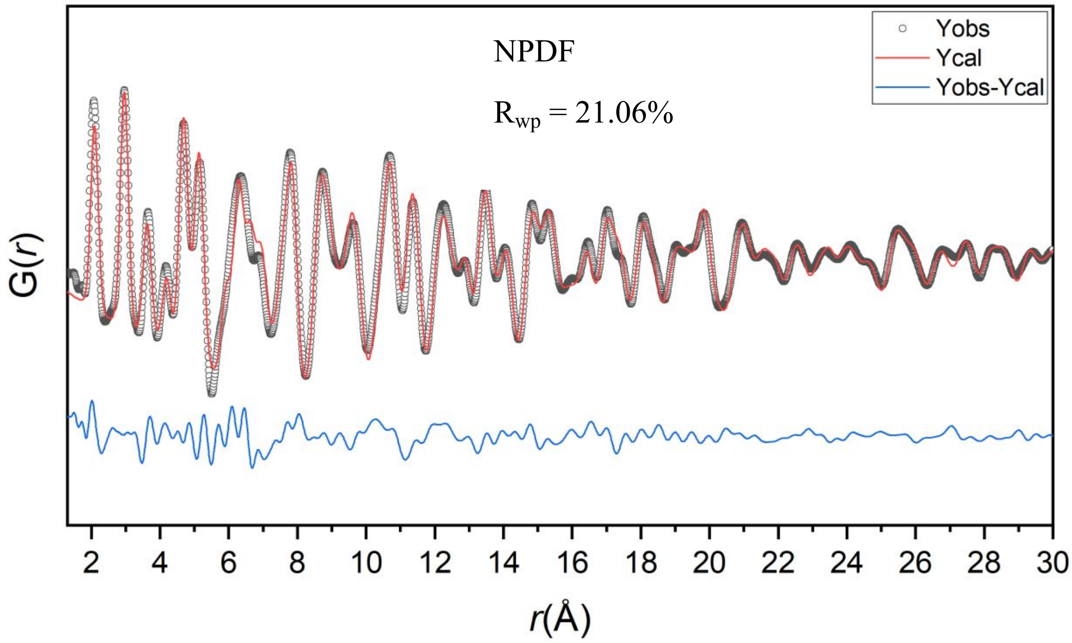

Figure S8. Small box PDF modelling of AMO-$Mg_{2.33}Al$ LDO SXRPD NPDF in the range of 1 – 30 Å using *Fm*3*m* rock salt model with tetrahedrally coordinated Al^{3+}.

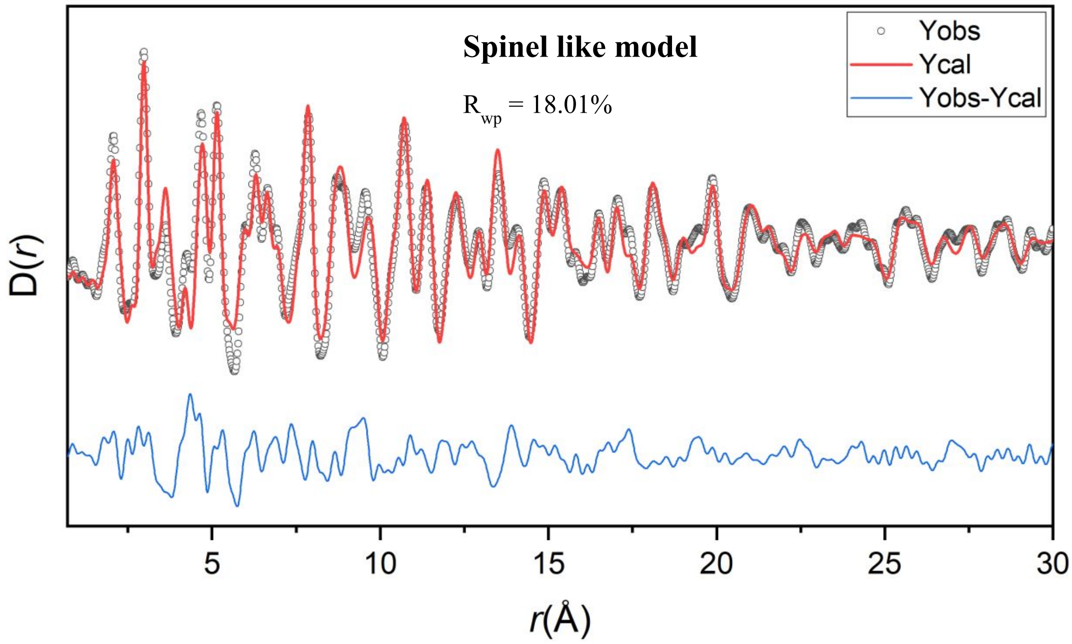

Figure S9. Small box PDF modelling of AMO-$Mg_{2.33}Al$ LDO SXRPD data using a modified spinel model proposed by Cherepanova *et al.*[1] in the range of 1 – 30 Å.

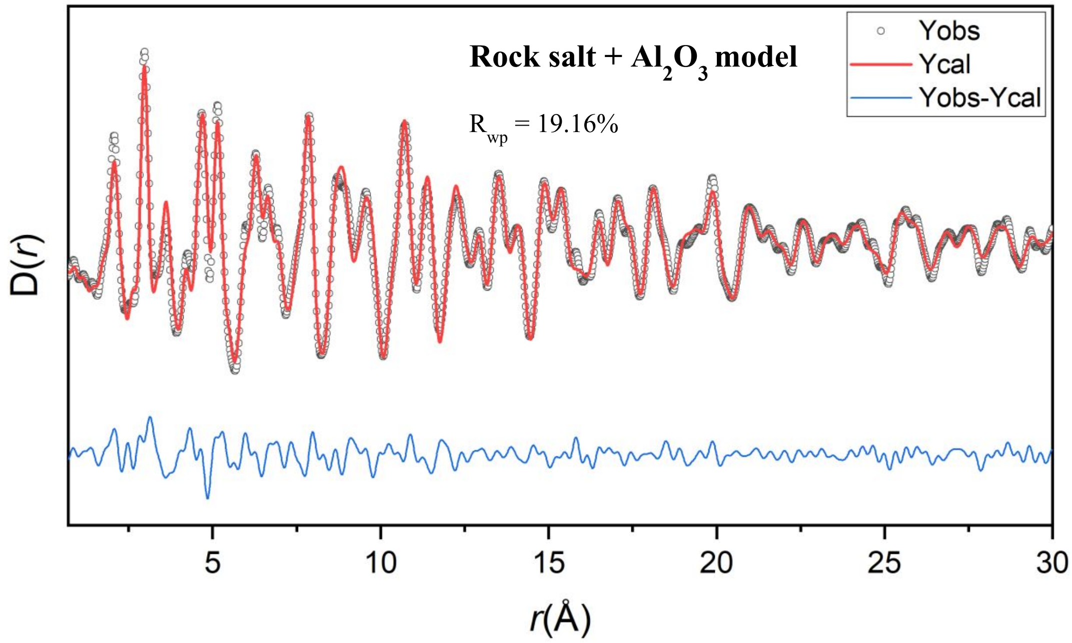

Figure S10. Small box PDF modelling of AMO-$Mg_{2.33}Al$ LDO XPDF data a two phase Rock salt and Al_2O_3 model proposed by Aramendia *et al.*[3] in the range of 1 – 30 Å.

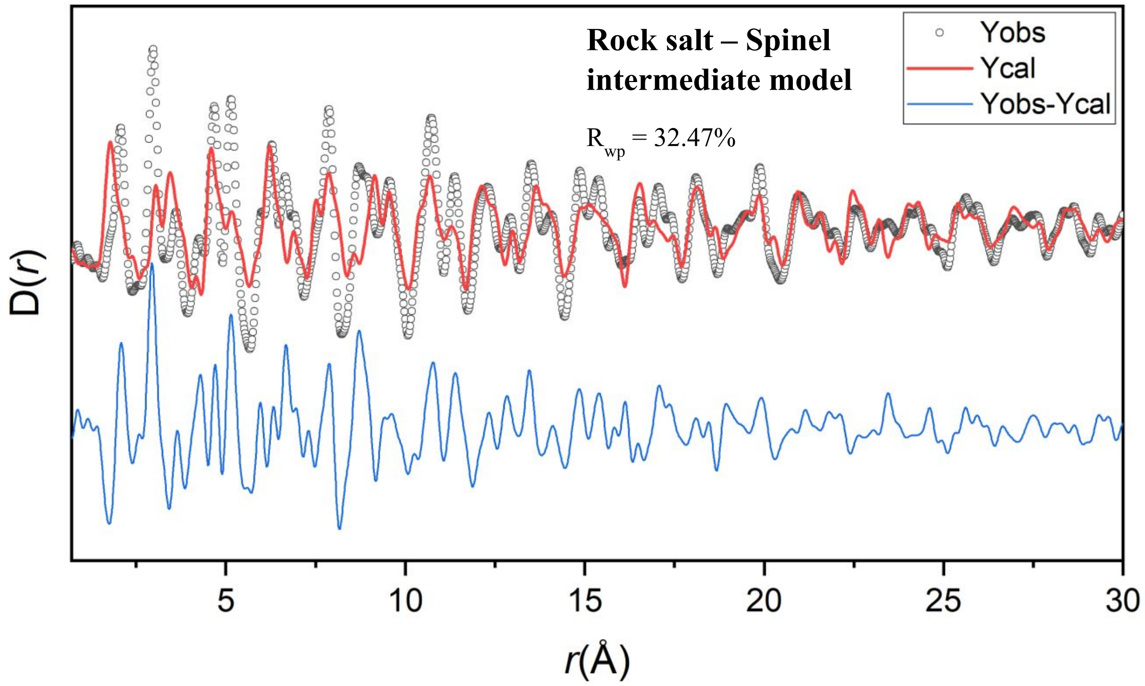

Figure S11. Small box PDF modelling of AMO-$Mg_{2.33}Al$ LDO XPDF data using a periclase – spinel intermediate model proposed by Johnsen *et al.*[2] in the range of 1 – 30 Å.

REFERENCES

(1) Cherepanova, S. V.; Leont'eva, N. N.; Arbuzov, A. B.; Drozdov, V. A.; Belskaya, O. B.; Antonicheva, N. V. Structure of oxides prepared by decomposition of layered double and hydroxides. *J Solid State Chem* **2015**, *225*, 417–426.
(2) Johnsen, R. E.; Norby, P. A Structural Study of Stacking Disorder in the Decomposition Oxide of MgAl Layered Double Hydroxide: A DIFFaX plus Analysis. *J Phys Chem C* **2009**, *113* (44), 19061–19066.
(3) Aramendía, M. A.; Borau, V.; Jiménez, C.; Marinas, J. M.; Ruiz, J. R.; Urbano, F. J. XRD and H MAS NMR spectroscopic study of mixed oxides obtained by calcination of layered-double hydroxides. *Mater Lett* **2000**, *46* (6), 309–314.
